# Cranioplastic Operations Following Loss of Bone Substance

**Published:** 1918-12

**Authors:** 


***Cranioplastic Operations Following a Loss of Bone Substance***. By Dr. H. Mayet. *Revue Interalliée pour les Mutilés de la Guerre*, June, 1918.

Dr. Mayet examines the three following points :

*a*) Can the loss of bony substance repair spontaneously?

*b*) Does cranioplasty present dangers?

*c*) Which are the most harmless cranioplastic operations, and which give the surest results?

*a*) Wounds of the skull seem to repair spontaneously, but Dr. Mayet thinks that, in most cases, it is only a fibrous substance and not a bony one that stops the wound. X-ray control applied in 21 cases by Dr. Mayet never revealed newly-made bony substance, even after 18 months or a year's time. Sometimes, however, it may be that some very small fragments of periosteum or dura-mater may remain in the wound. These fragments can regenerate bony substance and help in repairing the loss, especially if the wound is a small one. But these cases are rare. The actual method of cleansing the wounds, and excising widely all bruised tissues, lessens still further these chances of bone regeneration. Of course, the fibrous scar existing is always a weak point in the skull.

*b*) Dr. MAYET relates that the cranioplastic operations have made much progress since the beginning of the war. He considers them always as slight operations, but they must not be attempted too soon. It must be ascertained first that there is no quiescent infection, no adherences, no more splinters in the wound.

*c*) Many methods are in use for these operations. According to the nature of the repairing substance, the author distinguishes four cases :

1) Repairs may be made with a non-living substance : ivory, metal, lime-paste, or calcinated bone.

2) With cartilaginous grafts.

3) With osteoperiosteal grafts taken from other bones of the body.

4) With osteoperiosteal grafts taken in the neighborhood of the wound. By this method the graft continues to live and increase.

According to the last two methods, it is as if a lid were fixed upon the wound stopping it. According to the first two, the hole is filled up with the repairing substance. It may be feared that disturbances due to a compression of the brains might occur more frequently in those cases.

Statistics published by Villandre in the *Presse Médicale* (May 24, 1917) give the following proportions of excellent results : lime-paste, 50 per cent. ; cartilaginous cranioplasty, 96.8 per cent. ; grafts taken from the skull, 100 per cent.

